# Preparation and Characterization of Monoclonal Antibodies Against the Porcine Rotavirus VP6 Protein

**DOI:** 10.3390/vetsci12080710

**Published:** 2025-07-29

**Authors:** Botao Sun, Dingyi Mao, Jing Chen, Xiaoqing Bi, Linke Zou, Jishan Bai, Rongchao Liu, Ping Hao, Qi Wang, Linhan Zhong, Panchi Zhang, Bin Zhou

**Affiliations:** 1 MOE Joint International Research Laboratory of Animal Health and Food Safety, College of Veterinary Medicine, Nanjing Agricultural University, Nanjing 210095, China; 2024007018@stu.njau.edu.cn (B.S.); t2025065@stu.njau.cn (J.C.); 2024207017@stu.njau.edu.cn (X.B.); linkezou@stu.njau.edu.cn (L.Z.); baijishan@stu.njau.edu.cn (J.B.); 2022807132@stu.njau.edu.cn (R.L.); 2024207023@stu.njau.edu.cn (P.H.); 2023007092@stu.njau.edu.cn (Q.W.); zhonglinhan1115@163.com (L.Z.); 2024807252@stu.njau.edu.cn (P.Z.); 2 Jiangsu Institute of Parasitic Diseases, Wuxi 214000, China; maodingyi@jipd.com; 3 Key Laboratory of Animal Bacteriology, Ministry of Agriculture and Rural Affairs, Nanjing Agricultural University, Nanjing 210095, China; 4 College of Veterinary Medicine, Northeast Agricultural University, Harbin 150030, China; 5 Northeast Science Observation Station for Animal Pathogen Biology, Ministry of Agriculture and Rural Affairs, Harbin 150030, China

**Keywords:** Porcine Rotavirus, hybridoma technology, antibody specificity, B-cell epitopes, epitope conservation

## Abstract

Porcine Rotavirus (PoRV), a predominant causative agent of neonatal diarrhea in piglets, shares substantial genetic homology with human rotavirus and represents a considerable threat to both public health and the global swine industry in the absence of specific antiviral interventions. The VP6 protein, an internal capsid component, is characterized by exceptional sequence conservation and robust immunogenicity, rendering it an ideal candidate for viral genotyping and vaccine development. In the present study, the recombinant plasmid pET28a(+)-VP6 was engineered to facilitate the high-yield expression and purification of the VP6 antigen. BALB/c mice were immunized to generate monoclonal antibodies (mAbs) through hybridoma technology, and the antigenic specificity of the resulting mAbs was stringently validated. Subsequently, a panel of truncated protein constructs was designed to precisely map linear B-cell epitopes, followed by comparative conservation analysis across diverse PoRV strains. Functional validation demonstrated that all three mAbs exhibited high-affinity binding to VP6, with a peak detection titer of 1:3,000,000 and exclusive specificity toward PoRVA. These antibodies effectively recognized representative genotypes such as G3 and X1, while exhibiting no cross-reactivity with unrelated viral pathogens; however, their reactivity against other PoRV serogroups (e.g., types B and C) remains to be further elucidated. Epitope mapping identified two novel linear B-cell epitopes, ^128^YIKNWNLQNR^137^ and ^138^RQRTGFVFHK^147^, both displaying strong sequence conservation among circulating PoRV strains. Collectively, these findings provide a rigorous experimental framework for the functional dissection of VP6 and reinforce its potential as a valuable diagnostic and immunoprophylactic target in PoRV control strategies.

## Simple Summary

Porcine Rotavirus (PoRV), particularly genotype A, is globally prevalent and currently lacks effective antiviral therapies. Its segmented genome enables frequent genetic reassortment, posing substantial challenges for prevention and control. The development of effective vaccines is therefore imperative. VP6, a structurally conserved and highly immunogenic protein, represents an attractive target for vaccine development. In the present study, a monoclonal antibody (mAb) specifically targeting VP6 was generated, establishing a robust experimental foundation for improved PoRV diagnostics and vaccine research. This mAb demonstrated exceptional specificity against VP6, attaining a maximal detection titer of 1:3,000,000. Additionally, two previously uncharacterized B-cell epitopes were delineated, both exhibiting substantial conservation across diverse circulating PoRV strains. Collectively, these findings underscore the mAb’s significant potential as a tool for the advancement of VP6-based diagnostic methodologies and therapeutic interventions against PoRV.

## 1. Introduction

Rotavirus (RV) is a globally pervasive zoonotic pathogen, initially identified in diarrheic cattle in the United States in 1969 and subsequently disseminated worldwide [1]. In 1976, the International Committee on Taxonomy of Viruses designated this viral morphology as “RV” [2]. Rotavirus primarily infects a broad spectrum of mammalian hosts—including humans, bovines, ovines, canines, and swine—eliciting acute gastroenteritis. Its segmented double-stranded RNA genome facilitates frequent genetic reassortment, fostering the evolution of novel viral variants. This genomic plasticity markedly hinders the development of effective prophylactic measures and surveillance protocols, thereby amplifying both global economic losses and public health threats [3].

Currently, the Rotavirus Classification Working Group (RCWG) categorizes Rotavirus A (RVA) into GX-P[x]-Ix-Rx-Cx-Mx-Ax-Nx-Tx-Ex-Hx genotypes based on the open reading frames of its 11 genomic segments [4,5], which encode structural proteins (VP1, VP2, VP3, VP4, VP6, VP7) and non-structural proteins (NSP1–NSP6). The viral genome is encapsulated within a triple-layered capsid composed of VP2, VP6, and VP7. VP6 constitutes the intermediate layer surrounding VP2. In insect and plant cells, co-expression of VP2 and VP6 facilitates the assembly of double-layered viral particles [6]. VP6, a central structural protein, possesses high immunogenic potential and has been widely studied for its functional contributions to viral assembly and host immune modulation. Notably, recombinant virus-like particles incorporating VP2, VP6, and VP7 have demonstrated the capacity to elicit strong and protective immune responses in mouse models [7]. Moreover, VP6 is extensively employed in viral diagnostic platforms and fulfills pivotal roles in viral replication and protein biosynthesis.

Rotaviruses are taxonomically classified into ten distinct groups (A-J) based on VP6 antigenic determinants, among which RVA is the most prevalent and pathogenic [8]. In swine populations, Porcine Rotavirus A (PoRVA) constitutes a primary etiological agent of acute enteritis in suckling piglets under eight weeks of age, with morbidity rates ranging from 60% to 80% and mortality rates reaching 15–25%. This substantially compromises piglet viability and production efficiency. Furthermore, PoRVA frequently co-infects with other enteric viruses, such as Porcine Epidemic Diarrhea Virus (PEDV) and Transmissible Gastroenteritis Virus (TGEV), as well as bacterial pathogens such as *Escherichia coli* [9]. These co-infections exacerbate disease severity and transmission, resulting in significant economic losses across the swine industry [10]. Although prior research has reported the generation of monoclonal antibodies (mAbs) against VP6 for diagnostic applications and protective assessment, existing approaches are constrained by limited breadth and poor cross-reactivity across PoRVA subtypes [11]. Thus, the development of high-affinity, broadly reactive mAbs targeting VP6 is imperative to advance diagnostic capabilities and inform next-generation vaccine strategies.

Despite considerable progress in VP6-related research, there remains a pronounced deficit in the development of mAbs with diagnostic utility and therapeutic potential against PoRV—particularly those capable of targeting conserved epitopes with high specificity and efficacy. To bridge this gap, the present study endeavors to express and purify recombinant VP6 protein using a prokaryotic expression platform, followed by the induction of antigen-specific immune responses in BALB/c mice through a combined subcutaneous and intraperitoneal immunization regimen. High-affinity, VP6-specific mAbs will be screened via hybridoma technology. In parallel, epitope mapping will be conducted to delineate evolutionarily conserved B-cell epitopes recognized by these mAbs. In conclusion, this study seeks to establish a rigorous experimental framework, with the goal of providing a robust experimental foundation and theoretical support for the development of precise diagnostic approaches for PoRV and the optimization of targeted intervention strategies.

## 2. Materials and Methods

### 2.1. Experimental Material

PoRV was isolated and characterized by our laboratory, with partial cell lysate samples from PoRV-infected cells kindly provided by senior researcher Li Bin, Institute of Veterinary Medicine, Jiangsu Academy of Agricultural Sciences., Nanjing, China. JEV was isolated and identified by our laboratory. MA104 cells and mouse myeloma SP2/0 cells were maintained in our laboratory. Specific pathogen-free (SPF) BALB/c mice were procured from Changzhou Cavens Experimental Animal Co., Ltd., Changzhou, China. and housed at the Experimental Animal Center, Nanjing Agricultural University. All immunization procedures adhered to the institutional animal care and use guidelines (Animal Welfare and Ethics Approval No. NJAU.No20220706145). The pET-28a(+)-VP6 plasmid was synthesized by the GenScript Biotech Corporation., Nanjing, China.

### 2.2. Enzymes and Related Reagents

The prokaryotic expression vector pET-28a(+) was preserved in our laboratory. Competent *Escherichia coli* DH5α (Cat. No. TSC-C01) and BL21 (Cat. No. TSC-E01) were obtained from Beijing Tsingke Biotechnology Co., Ltd., Beijing, China. Green Taq Mix (Cat. No. P131-01), a 180 kDa prestained protein marker (Cat. No. MP102-01), the BCA Protein Assay Kit (Cat. No. E112-01), and the High-Sensitivity ECL Chemiluminescence Detection Kit (Cat. No. E412-01) were purchased from Nanjing Novizan Medical Technology Co., Ltd., Nanjing, China. PrimeSTAR high-fidelity PCR polymerase (Cat. No. R040A), restriction endonucleases BamHI (Cat. No. 1010S) and XhoI (Cat. No. 1094S), DNA marker (Cat. No. 3427Q/3582Q), 10 × nucleic acid loading buffer (Cat. No. 9157), T4 DNA ligase (Cat. No. 2011A), and the DNA Gel Extraction Kit (Cat. No. 9762) were sourced from TaKaRa Bio, Inc. (Beijing, China). The 180 kDa three-color prestained protein marker (Cat. No. WJ103) and 12.5% PAGE Gel Rapid Preparation Kit (Cat. No. PG113) were purchased from Shanghai Yamei Biotechnology Co., Ltd., Shanghai, China. The plasmid extraction kit (Cat. No. D6943-01) was from Omega Bio-Tek., Nanjing, China. PoRV-positive serum was kindly provided by New Hope Liuhe Co., Ltd., Chengdu, China. HRP-conjugated goat anti-mouse IgG (Cat. No. SA00001-1), FITC-conjugated goat anti-mouse IgG (Cat. No. SA00003-1), anti-His-tag antibody (Cat. No. 66005-1-Ig), and the Mouse Monoclonal Antibody Isotyping Kit (Cat. No. PK20003) were obtained from Proteintech Group. HRP-conjugated goat anti-pig IgG (Cat. No. ab6915) was purchased from Abcam.

### 2.3. Truncated Expression of Recombinant Gene pET-28a(+)-VP6

The full-length VP6 gene sequence was obtained using RVA/Porcine-tc/KOR/174-1/2006/G8P (GenBank No. MF940552.1) as the template. Hydrophobicity analysis was conducted using TMHMM-2.0, and the three-dimensional structural model was constructed via SWISS-MODEL homology modeling. Based on antigenicity prediction and sequence conservation, the N-terminal 2–178 aa region was selected as the target fragment. Specific primers containing BamHI and XhoI restriction sites were designed and synthesized by GenScript Biotech (Table S1). PCR amplification and double digestion verification were performed for recombinant plasmid construction.

### 2.4. Expression and Purification of VP6 Recombinant Protein

The sequence-verified plasmid was extracted and subsequently transformed into *E. coli* BL21(DE3) competent cells. Upon optimizing the induction parameters, VP6 was abundantly expressed as inclusion bodies, which were subsequently subjected to partial denaturation and refolding protocols. The recombinant protein was then purified via nickel–nitrilotriacetic acid (Ni-NTA) affinity chromatography. The purity and identity of the isolated VP6 protein were validated through SDS-PAGE and Western blot (WB) analyses.

### 2.5. Immunization of Mice

Four female BALB/c mice (6–8 weeks) were immunized with emulsified VP6 protein according to the protocol in Table S2 [12]. One mouse received PBS emulsified with Freund’s complete adjuvant as a control. After the third immunization, blood was collected via orbital sampling to determine antibody titers for booster immunization and cell fusion preparation.

### 2.6. Cell Fusion and Subcloning

An indirect enzyme-linked immunosorbent assay (ELISA) was established by optimizing the coating concentrations, blocking times, and antibody dilutions, using a P/N ratio ≥2.1 as the positivity threshold for subcloning and detection [13]. SP2/0 cells were thawed, and splenocytes were harvested 3 days post-booster immunization. Cell fusion was performed at a 1:5 ratio (SP2/0–splenocytes) using PEG 4000(Nanjing, China) [11]. Hybridoma subcloning was conducted using the ELISA screening protocol, and stable antibody-secreting cell lines were expanded for ascites production.

Following sensitization, ~2×10^6^ hybridoma cells were intraperitoneally injected into 6–8-week-old BALB/c mice. Ascites fluid was collected and stored at −20 °C and then purified via IgG affinity chromatography. Antibody titers and subtypes were determined by ELISA; specificity was validated by WB; and reactivity against the virus was confirmed by immunofluorescence assay (IFA).

### 2.7. Identification of Monoclonal Antibody Specificity

MA104 cells were infected with PoRV at a multiplicity of infection (MOI) of 1. Upon the onset of distinct cytopathic effects (CPE), IFA was conducted utilizing the VP6-specific monoclonal antibody (mAb) diluted at 1:100 as the primary antibody and a goat anti-mouse FITC-conjugated antibody as the secondary to evaluate antibody specificity and sensitivity. BHK cells infected with JEV were used as negative controls, and their reactivity was assessed using both the VP6 mAb and a JEV-E-specific antibody.

In parallel, MA104 cells were infected with PoRV G3 and X1 genotypes, while BHK cells were infected with JEV. Uninfected cells served as negative controls. Following cell lysis and protein extraction, Western blotting was performed using the VP6 mAb as the primary antibody and HRP-conjugated secondary antibodies. To further substantiate the assay’s specificity, heterologous antibodies and the JEV-NS5 antibody were employed as negative controls.

### 2.8. Construction of VP6 Protein Truncation Mutants

Epitope identification defines critical antigenic regions recognized by mAbs, underpinning affinity optimization, specificity enhancement, and broad-spectrum antiviral development. Following methodologies for truncation variant construction [14], primers were designed using a dichotomy approach to amplify the 1–97 aa (F1) and 87–174 aa (F2) fragments, with a 10 aa overlap. Using pET-28a as a vector and BamHI/XhoI sites, recombinant plasmids were constructed and sequenced (Sangon Biotech, Shanghai, China). Considering typical epitope lengths (6–15 aa), a 10 aa step size balanced mapping efficiency and resolution [15]. Subsequent truncation primers were designed with sequential 10 aa deletions, yielding nine primer pairs (Table S3).

### 2.9. Epitope Identification and Conservation Analysis

Truncated proteins were expressed, and WB was performed using ascites from mAbs 2E7, 2E4, and 3G6 to identify epitope-containing regions.

Full-length VP6 sequences from eight prevalent PoRV strains (Accession Nos. AAB41105.1, AER25319.1, ANQ45164.1, AOW44086.1, AXS77346.1, AYA93307.1, AYA93308.1, AYA93321.1, AYA93335.1) were retrieved from the NCBI and aligned with the VP6 variant (AXF43000.1) using MegAlign Pro 17.1 to evaluate epitope conservation.

The procedural workflow for monoclonal antibody production is illustrated schematically. Please refer to Supplementary Figure S1 for a detailed flowchart.

## 3. Results

### 3.1. Construction, Expression, and Purification of Recombinant pET-28a(+)-VP6

**Figure 1:**
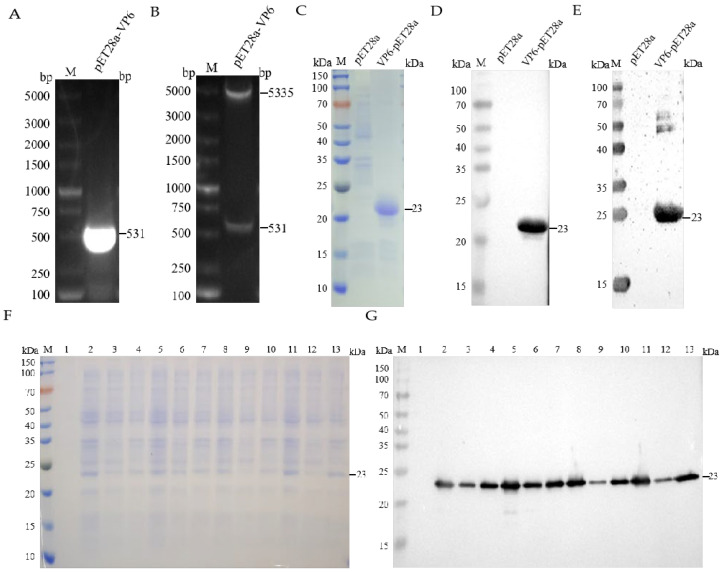
Expression and purification of recombinant protein pET-28a(+)-VP6. (**A**) Results of VP6 gene amplification. (**B**) Restriction enzyme digestion identification of recombinant plasmid pET28a(+)-VP6. (**C**) Results of SDS-PAGE for protein-induced expression. (**D**) Results of WB for protein-induced expression. (**E**) Verification of recombinant protein pET-28a(+)-VP6 by SDS-PAGE. (**F**) SDS-PAGE results of induced expression of pET28a(+)-VP6 protein. Lane 1: uninduced bacteria; lanes 2–4: whole bacteria, supernatant, and precipitate induced with 0.5 mM IPTG at 16 °C; lanes 5–7: whole bacteria, supernatant, and precipitate induced with 1 mM IPTG at 16 °C; lanes 8–10: whole bacteria, supernatant, and precipitate induced with 0.5 mM IPTG at 37 °C; lanes 11–13: whole bacteria, supernatant, and precipitate induced with 1 mM IPTG at 37 °C. (**G**) WB results of induced expression of pET28a(+)-VP6 protein. The lanes are the same as in (**E**).

The complete genomic sequence of the RVA/Porcine-tc/KOR/174-1/2006/G8P strain (Accession No. MF940552.1) was retrieved from GenBank. Transmembrane domain analysis using TMHMM-2.0 predicted no transmembrane regions within VP6 (Figure S2). Structural modeling based on the predicted tertiary conformation (Figure S3) guided the selection of an evolutionarily conserved and immunodominant region (2–178 aa) for recombinant expression. The results of target gene amplification and double restriction enzyme digestion confirmed the successful integration of the target gene into the pET-28a(+) vector (Figure 1A,B). SDS-PAGE and WB analyses confirmed the robust expression of the recombinant VP6 protein in the form of inclusion bodies following IPTG induction (1 mM, 37 °C) (Figure 1F,G). Subsequent purification via Ni-NTA affinity chromatography yielded a distinct, homogeneous protein band, which was concentrated to 2.09 mg/mL using sucrose gradient ultrafiltration (Figure 1C). WB analysis utilizing an anti-His tag monoclonal antibody and hyperimmune serum derived from Group A rotavirus-infected swine validated the immunoreactivity and antigenic specificity of the purified VP6 protein (Figure 1D,E).

### 3.2. Generation and Characterization of VP6-Specific Monoclonal Antibodies

**Figure 2:**
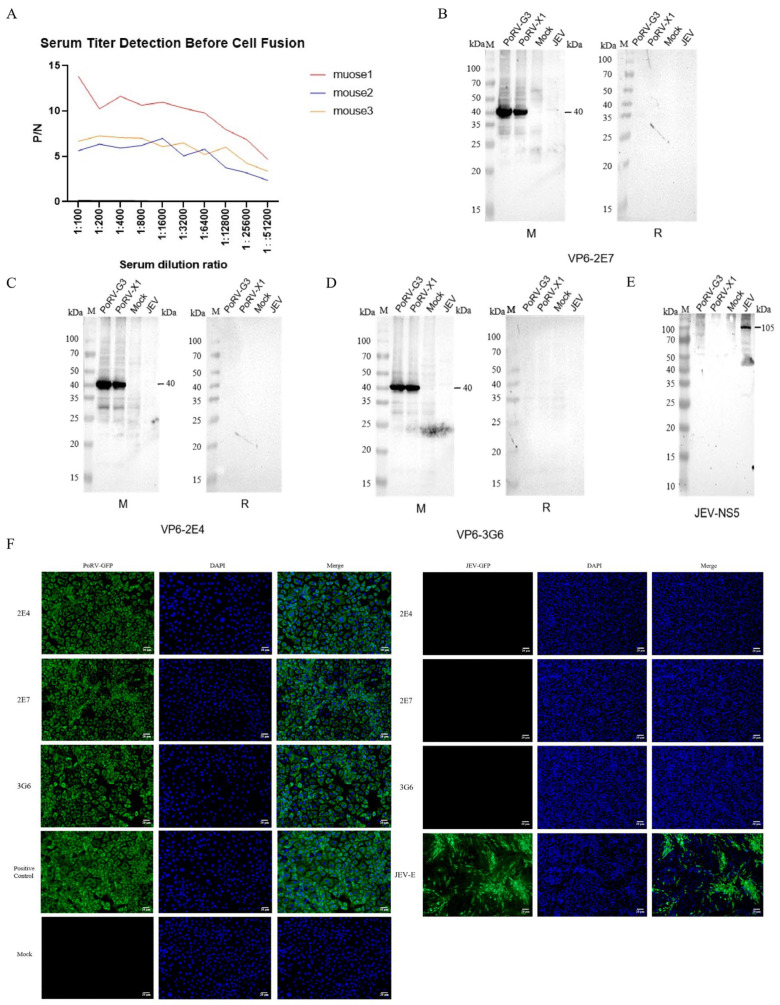
Identification of monoclonal antibodies. (**A**) Antibody levels in three mice after immunization. (**B**–**E**) Specific identification of VP6 mAbs. M: 250 kDa protein marker. PoRV-G3: lysate of cells infected with PoRV-G3. PoRV-X1: lysate of cells infected with PoRV-X1. Mock: lysate of uninfected cells. JEV: lysate of cells infected with Japanese Encephalitis Virus. M refers to mouse-derived secondary antibody, and R refers to rabbit-derived secondary antibody. (**F**) An indirect immunofluorescence assay was performed to determine the reactivity of supernatants from three hybridoma cell lines with both PoRV-infected MA104 cells and JEV-infected BHK cells. Proteins in virus-infected cells were labeled with green fluorescence.

The optimization of the indirect ELISA established the following optimal parameters: carbonate buffer coating, antigen concentration at 200 ng/well, blocking at 37 °C for 2 h, and a secondary antibody dilution of 1:5000. Among the immunized mice, Mouse 1 exhibited the most potent humoral response and was subsequently selected for hybridoma production (Figure 2A). Following four cycles of subcloning, three stable hybridoma cell lines—2E7, 2E4, and 3G6—were established, all capable of the continuous secretion of VP6-specific monoclonal antibodies. Isotype characterization revealed that 2E7 secreted IgG2a with a kappa light chain, demonstrating a titer >1:320,000, while 2E4 and 3G6 produced IgG1 isotypes with kappa light chains, each exceeding titers of 1:3,000,000.

These monoclonal antibodies exhibited exceptional specificity and high-affinity binding, forming a robust foundation for the development of precision diagnostics, the differential detection of co-infections, and advanced immunoassays. WB assays confirmed that all three mAbs specifically recognized the PoRV G3 and X1 genotypes, without cross-reactivity toward heterologous secondary antibodies (Figure 2B–D). Specificity was further substantiated using the JEV-NS5 protein as a negative control (Figure 2E). Consistently, the IFA results corroborated the WB findings, wherein the hybridoma supernatants, diluted at 1:100, retained antigen-binding capabilities in PoRV-infected MA104 cells and exhibited no cross-reactivity with other viruses, such as JEV (Figure 2F).

### 3.3. Construction of VP6 Truncation Variants and Epitope Mapping

**Figure 3:**
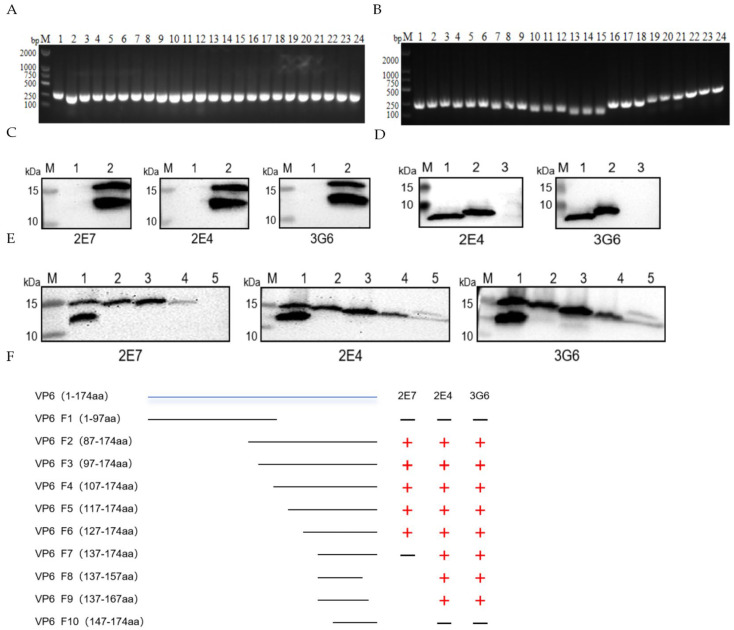
Identification of B-cell determinants of mAbs specific to VP6 monoclonal antibodies. (**A**) Construction of VP6 truncation mutants by dichotomy. Lanes 1-12: F1; 13-24: F2. (**B**) PCR amplification of VP6 truncation mutants in the last two rounds. Lanes 1-3: F8; 4-6: F9; 7-9: F10; 10-12: F7; 13-15: F6; 16-18: F5; 19-21: F4; 22-24: F3. (**C**) WB analysis of first-round truncation mutants with antibodies. Lane 1: F1-expressed protein; 2: F2-expressed protein. (**D**) Epitope mapping of 2E4 and 3G6 (antibody incubation). Lane 1: F8-expressed protein; 2: F9; 3: F10. (**E**) Epitope mapping of 2E7 (antibody incubation). Lane 1: F3-expressed protein; 2: F4; 3: F5; 4: F6; 5: F7. (**F**) Flowchart of truncation construct construction.

A bifurcated truncation strategy was employed to engineer polypeptide fragments spanning amino acids 1–97 and 87–174. Recombinant constructs were successfully assembled and authenticated via Sanger sequencing (Figure 3A). WB analysis demonstrated that all three monoclonal antibodies recognized epitopes localized within the 98–174-amino-acid region (Figure 3C). Subsequently, systematic internal deletions were implemented across this region by sequentially excising 10-amino-acid segments. Each deletion variant was cloned in triplicate, and clones displaying consistent electrophoretic characteristics were selected for commercial sequencing (Sangon Biotech, Shanghai, China) (Figure 3B). The comprehensive epitope mapping workflow is depicted in Figure 3F. Epitope identification revealed that monoclonal antibody 2E7 specifically bound to residues 128–137 (YIKNWNLQNR) (Figure 3E), whereas antibodies 2E4 and 3G6 recognized a contiguous yet distinct epitope encompassing residues 138–147 (RQRTGFVFHK) (Figure 3D).

### 3.4. Epitope Conservation Analysis

**Figure 4:**
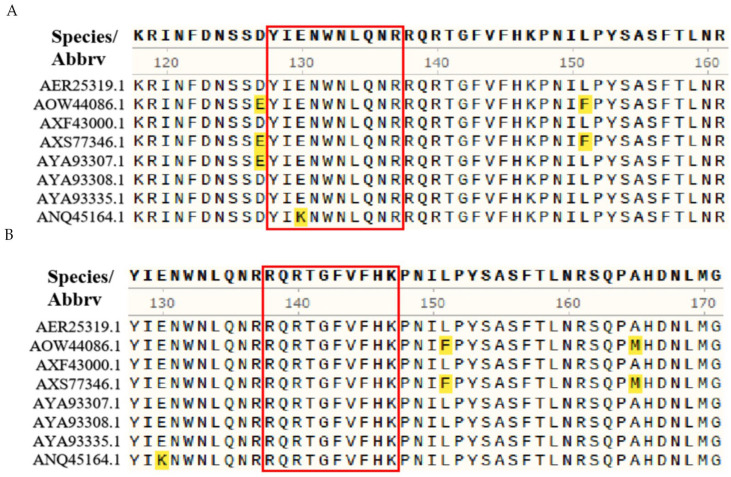
Epitope conservation analysis. (**A**) Multiple sequence alignment of the 2E7 epitope ^128^YIKNWNLQNR^137^ using MegAlign. (**B**) Multiple sequence alignment of the 2E4/3G6 epitope ^138^RQRTGFVFHK^147^ using MegAlign. Note: The region demarcated by the red frame in the figure corresponds to the epitope identified by the antibody, while the highlighted region denotes the differential segment in the sequence alignment.

Multiple sequence alignment using MegAlign revealed that, within epitope ^128^YIKNWNLQNR^137^, residue K130 was substituted with E130 in all analyzed strains except AXF43000.1, while the remaining residues were highly conserved across strains (Figure 4A). In contrast, the epitope ^138^RQRTGFVFHK^147^ was completely conserved among the eight representative Porcine Rotavirus isolates evaluated (Figure 4B).

## 4. Discussion

RV, a member of the Reoviridae family, constitutes a major causative agent of viral gastroenteritis in both young children and livestock worldwide [16,17]. First identified in swine feces in 1975 [18], PoRV is primarily transmitted via the fecal–oral route and causes clinical symptoms such as vomiting, anorexia, dehydration, and severe diarrhea in neonatal piglets, often leading to high morbidity and significant mortality [8]. Endemic diarrhea in piglets continues to impose considerable economic burdens on the swine industry, particularly in China. Contemporary surveillance implicates PoRVA as the predominant viral pathogen responsible for enteric disease in suckling and weaned piglets [8,19]. Furthermore, PoRVA exhibits zoonotic potential, with increasing evidence of cross-species transmission to humans and other economically critical livestock. For instance, epidemiological studies in India have revealed widespread PoRVA dissemination among pig populations, and the genetic characterization of VP7, NSP3, and NSP4 genes underscores its interspecies transmissibility [20]. The close sequence homology between PoRV and human RV strains suggests that the mAbs developed in this study may serve as diagnostic or therapeutic tools in a One Health context—facilitating rapid detection across host species and aiding in integrated control strategies. Notably, the conservation of the identified epitopes may offer a foundation for universal cross-species diagnostics, minimizing missed detections in public health monitoring programs. In China, PoRVA is consistently ranked as a leading etiological agent of piglet diarrhea, second only to Porcine Epidemic Diarrhea Virus (PEDV). Phylogenetic analyses of VP6 genes from Shandong strains revealed five distinct group A RV genotypes [9], underscoring their broad distribution and persistent threat to China’s swine sector [19]. Despite the disease burden, there are currently no effective antiviral treatments available for PoRV, rendering immunoprophylaxis the primary strategy to mitigate viral spread and reduce the clinical incidence [21,22,23,24]. Hence, the development of efficacious vaccines and precise, field-deployable diagnostic tools is critical to improve disease surveillance and ensure timely outbreak control [25,26].

VP6, the most prevalent genotype circulating in China, is characterized by extensive interstrain conservation, prominent immunogenicity, and unique strain-specific antigenic regions [27]. As the first RV protein to bridge immunoreactivity and genetic variability, VP6 has played a central role in rotavirus taxonomy and is a prime target for diagnostic and vaccine development efforts [28]. Owing to its high expression levels, structural integrity, and immunological prominence, VP6 is ideal for functional analyses and classification-based studies [29,30,31].

This study hypothesized that “monoclonal antibodies generated against conserved VP6 epitopes can specifically recognize PoRVA and thereby serve as viable tools for diagnostics and vaccine design.” Supporting this premise, we successfully produced high-affinity, VP6-specific mAbs with demonstrable specificity and no cross-reactivity to non-rotaviral pathogens. Currently, there is limited research on the VP6 protein of PoRVA and its monoclonal antibodies [32,33]. Despite its significance, research on PoRVA VP6-specific mAbs remains limited. For example, Badillo-Godinez et al. demonstrated that the parenteral administration of VP6 targeting DEC-205 elicited mucosal protection, whereas Atta Muhammad Memon et al. developed VP6-targeted antibodies for antigen capture ELISA without evaluating interstrain cross-reactivity. Notably, the three monoclonal antibodies (mAbs) developed in this study demonstrated high specificity for PoRVA, exhibiting no cross-reactivity with heterologous non-rotaviral pathogens. This result robustly substantiates the hypothesis that VP6 represents a highly specific and diagnostically viable antigenic target for PoRVA detection. For instance, Kohli et al. utilized pepscan technology to identify four antigenic domains on bovine RV VP6, while others characterized five linear B-cell epitopes via truncated expression and synthetic peptide mapping [34]. Analogously, preceding investigations utilized truncated protein expression in conjunction with synthetic peptide methodologies to delineate five linear antigenic epitopes positioned at amino acid residues 6–20, 96–110, 134–144, 302–310, and 361–372 [35]. Nevertheless, the limited conservation of specific epitopes, such as those characterized in the 1E5 reference strain, may undermine diagnostic specificity [35]. Additionally, a B-cell epitope encompassing residues 289–302 was identified through the use of overlapping synthetic peptides [27]. In this study, two novel linear B-cell epitopes (^128^YIKNWNLQNR^137^ and ^138^RQRTGFVFHK^147^) within the VP6 protein were elucidated. Conservation analysis revealed that these epitopes are highly conserved among circulating PoRVA strains, underscoring their broad-spectrum diagnostic potential. These findings align with the hypothesis that VP6 conservation enables cross-genotypic detection. While epitope mapping via truncated protein constructs is efficient, technically straightforward, and cost-effective, it is inherently constrained in identifying conformational epitopes, as improper folding may result in epitope loss. Moreover, limitations in terms of expression yield and mapping resolution necessitate integration with complementary approaches to improve the analytical precision. Accordingly, iterative methodological optimization remains critical for the advancement of epitope-based diagnostic platforms and vaccine strategies.

In conclusion, this study successfully generated high-affinity and highly specific monoclonal antibodies against PoRVA VP6 through a prokaryotic expression platform. Two novel, evolutionarily conserved B-cell epitopes were delineated, furnishing a critical theoretical foundation for the development of VP6-oriented diagnostic assays and vaccine candidates. Nonetheless, certain limitations should be acknowledged: specificity assessments were restricted to prototype PoRVA strains and a limited subset of serotypes, with no validation performed against a broader spectrum of emergent variants or clinical isolates, potentially limiting translational applicability. Additionally, the functional activity of the mAbs was evaluated exclusively via Western blotting and indirect ELISA, without in vivo assessment of their neutralizing efficacy; therefore, their neutralizing potential in vivo warrants further investigation. Subsequent investigations will prioritize the elucidation of cross-reactivity profiles against emerging PoRV genotypes (e.g., G9, G12) and geographically diverse clinical strains using molecular virology methodologies to substantiate their utility in epidemiological monitoring. Capitalizing on the conserved nature of the identified epitopes, a double-antibody sandwich ELISA employing these mAbs as capture antibodies will be established and integrated with existing PEDV detection platforms to facilitate rapid, dual-pathogen diagnostic capabilities for piglet enteric diseases. Furthermore, targeting conserved VP6 determinants to enhance diagnostic sensitivity and specificity will bolster early pathogen detection, molecular surveillance, vaccine innovation, and point-of-care diagnostics, thereby contributing substantial practical value to swine health management and public health preparedness.

## 5. Conclusions

Overall, this study successfully harnessed a prokaryotic expression system to produce and purify the VP6 protein of PoRV, laying the groundwork for the generation of three mAbs exhibiting superior specificity and sensitivity. These mAbs effectively recognized VP6 without eliciting non-specific interactions, underscoring their diagnostic potential. Through systematic epitope mapping, two previously uncharacterized linear B-cell epitopes (^128^YIKNWNLQNR^137^ and ^138^RQRTGFVFHK^147^) were identified, both highly conserved among diverse circulating PoRVA strains. The elucidation of these conserved epitopes and the development of corresponding high-affinity mAbs provide robust molecular tools and conceptual insights that will advance rapid diagnostic assay development, facilitate epidemiological monitoring, and inform the strategic design of VP6-based vaccines, thereby contributing substantially to PoRV prevention and control efforts.

## Data Availability

No new data were created or analyzed in this study. Data sharing is not applicable to this article.

## Supplementary Materials

The following supporting information can be downloaded at: https://www.mdpi.com/article/10.3390/vetsci12080710/s1, Figure S1: Flow chart of monoclonal antibody preparation; Figure S2: Prediction of the transmembrane domain of the VP6 gene; Figure S3: Prediction of the 3D modeling structure of the VP6 protein; Table S1: Specific Primers for VP6 Gene Amplification; Table S2: Immunization Protocol (Note: SC: Subcutaneous Injection; IP: Intraperitoneal Injection.); Table S3: Specific Primers for VP6 Gene Amplification.
