# Supplementary figures and images for: Preparation and Characterization of Monoclonal Antibodies Against the Porcine Rotavirus VP6 Protein
Source: Vet Sci. 2025 Jul 29;12(8):710. doi: 10.3390/vetsci12080710 (PMC12390022; doi:10.3390/vetsci12080710)

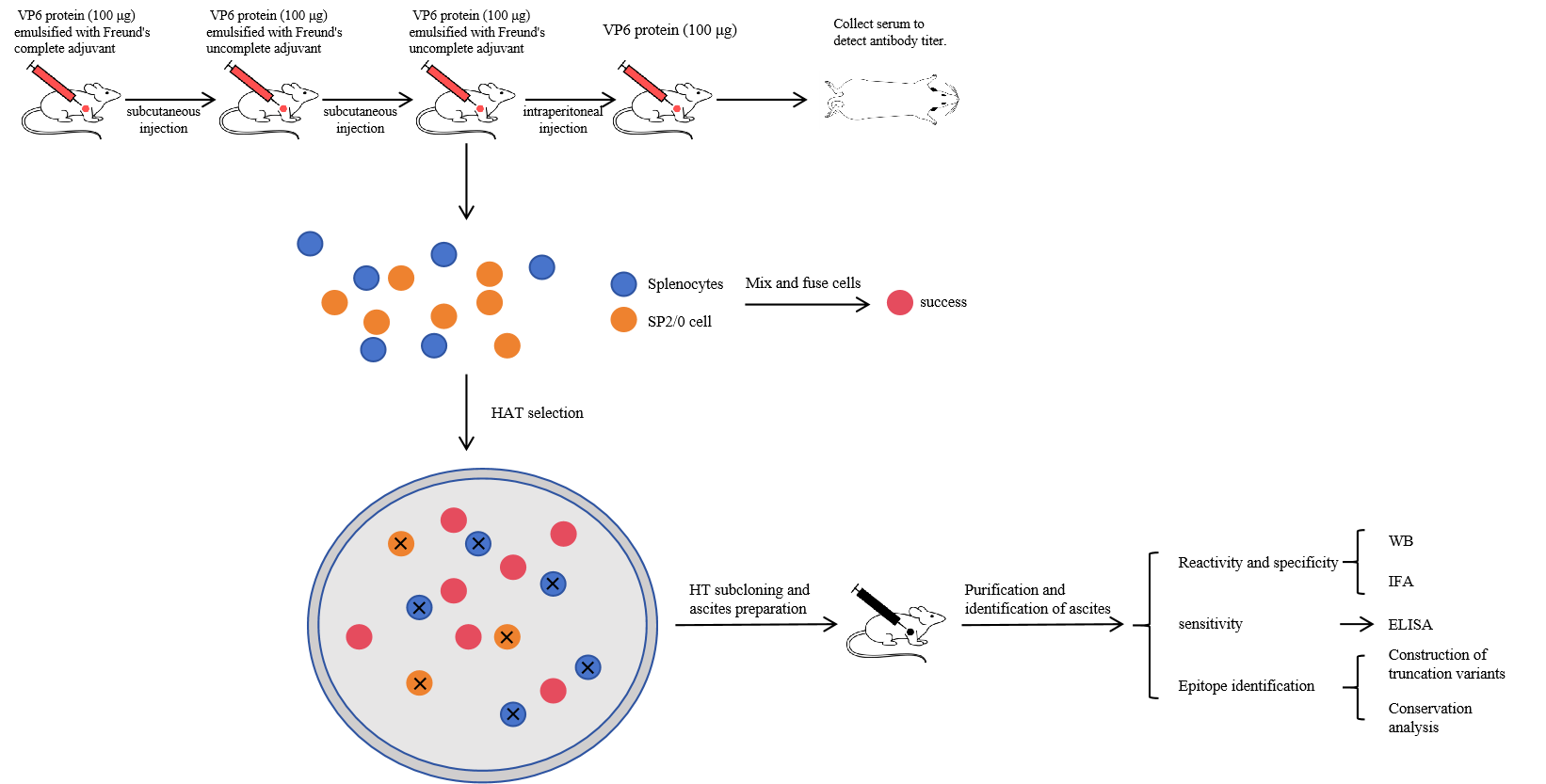

Supplement: Supplementary file 1 [file vetsci-12-00710-s001.zip › Fig-S1.tif]

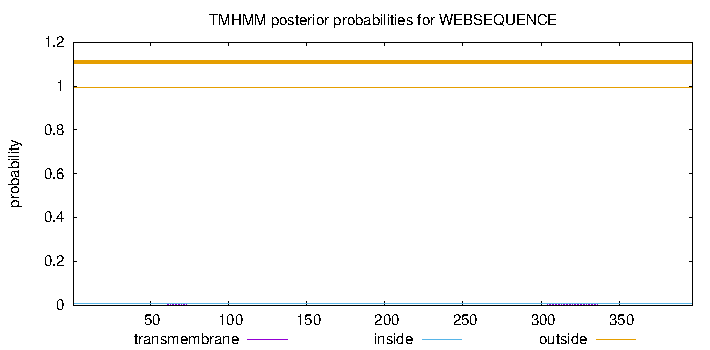

Supplement: Supplementary file 1 [file vetsci-12-00710-s001.zip › Fig-S2.tif]

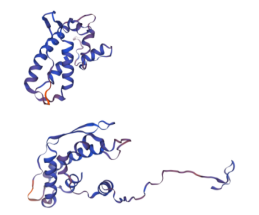

Supplement: Supplementary file 1 [file vetsci-12-00710-s001.zip › Fig-S3.tif]
